# The Spatial Correlations of Health Resource Agglomeration Capacities and Their Influencing Factors: Evidence from China

**DOI:** 10.3390/ijerph17228705

**Published:** 2020-11-23

**Authors:** Qingbin Guo, Kang Luo, Ruodi Hu

**Affiliations:** 1 School of Economics, Hainan University, Haikou 570228, China; 994076@hainanu.edu.cn (Q.G.); 20182581310092@hainanu.edu.cn (R.H.); 2 School of Economics & Management, Nanchang University, Nanchang 330031, China

**Keywords:** health resource agglomeration capacity, spatial correlation, influencing factors, China

## Abstract

We measured the health resource agglomeration capacities of 31 Chinese provinces (or municipalities) in 2004–2018 based on the entropy weight method. Using a modified spatial gravity model, we constructed and analyzed the spatial correlation network of these health resource agglomeration capacities and their influencing factors through social network analysis. We found that: (i) China’s health resource agglomeration capacity had a gradual strengthening trend, with capacity weakening from east to west (strongest in the eastern region, second strongest in the central region, and weakest in the western region). (ii) The spatial network of such capacities became more densely connected, and the network density and level (efficiency) showed an upward (downward) trend. (iii) In terms of centrality, the high-ranking provinces (or municipalities) were Beijing, Shanghai, Jiangsu, Zhejiang, Guangdong, Shandong, Hunan, Hubei, Fujian, Anhui, Jiangxi, and Tianjin, while the low-ranking were Tibet, Qinghai, Gansu, Ningxia, Inner Mongolia, Heilongjiang, Yunnan, Guizhou, Xinjiang, Hainan, Shaanxi, and Shanxi. (iv) Block 1 (eight provinces or municipalities), including Beijing, Tianjin, and Hebei, had a “net spillover” effect in the spatial network of health resource agglomeration capacities; Block 2, (seven provinces or municipalities), including Shanghai, Jiangsu, and Zhejiang, had a “bidirectional spillover” effect in the spatial network; Block 3 (seven provinces or municipalities), including Anhui, Hubei, and Hunan, had a “mediator” effect in the network; and Block 4, (nine provinces or municipalities), including Sichuan, Guizhou, and Tibet, had a “net beneficial” effect in the network. (v) The economic development, urbanization wage, and financial health expenditure levels, and population size had significant positive correlations with the spatial network of health resource agglomeration capacities. Policy recommendations to enhance the radiating role of health resources in core provinces (or municipalities), rationally allocate health resources, and transform ideas to support public health resource services were provided.

## 1. Introduction

In early 2020, the coronavirus disease 2019 (COVID-19) broke out in China. As of September 2020, the cumulative number of confirmed cases had exceeded 85,000 in China, challenging people’s lives and health in an unprecedented manner. Health, as a basic human aspiration, is fundamental for the comprehensive development of human beings. The guarantee of an individual’s right to health is not only related to the well-being of the individual but also affects the sustained development of society and economy. The Outline Development Plan of Healthy China 2030 was examined and approved at a meeting on 26 August 2016. President Xi Jinping, who is also the general secretary of the Communist Party of China (CPC), stressed at the meeting that the health of the people is a priority for development to a strategic position. Considering the fundamental role of health resources in promoting all-round development of the economy, society, and human beings, it has become an important symbol of social justice for the state to maintain equal access to health resources and services, and it has become incumbent upon the government to provide basic health resource services and ensure that “everyone has access to basic medical and health services. “At present, China has established a multi-level medical security system including basic medical insurance, critical illness insurance, supplementary medical insurance for employees, medical subsidies for civil servants, medical assistance, and commercial health insurance. According to data from the National Medical Insurance Administration, the number of people participating in China’s basic medical insurance in 2019 reached 1.354 billion, and the participation rate has stabilized at more than 95%.

Health resource agglomeration capacity refers to the ability of a region to attract various health resources from surrounding regions (even the whole country) to concentrate in the region, thereby creating value-added and spillover effects. It mainly includes human, material, and financial resources related to health, and is mainly measured using indicators, such as the number of medical and health institutions, hospital beds, and certified physicians. China’s research on health resource agglomeration capacity started later than that of other countries, but it has been developing relatively fast. Specifically, the Severe Acute Respiratory Syndrome (SARS) outbreak in 2003 prompted the rethinking of a nationwide construction of public health systems. However, factors, such as imbalances in regional economic development, differences between central and local fiscal investments, income disparities, and regional segmentation of health resources, have led to large differences in the health resource agglomeration capacities of different regions [1]. To address the spatial disparity in health resource agglomeration capacities, the Ministry of Health, Ministry of Finance, and National Health and Family Planning Commission (formerly the National Population and Family Planning Commission) put forward the *Opinions on Promoting the Gradual Equalization of Basic Public Health Services* in 2009, aiming to narrow the regional and urban–rural disparities in health resource agglomeration capacities. Existing studies on health resource agglomeration capacities are either been conducted at the national level using national data [2,3,4], or at the inter-provincial level using data from 31 provinces (or municipalities) across the country [5], or at the provincial (or municipal) level using data of a particular province (or municipality) [6,7]; or at the regional level [8]. Studies use panel data [9], cross-sectional data [10], or time series data [11]. In terms of the research methods, most researchers use models (e.g., logit model) for analysis [12], whereas a few researchers use the questionnaire method [13,14], entropy-weighted technique for preference by similarity to ideal solution (TOPSIS) [15], and indicator metrics [16].

In the context of regional integration, the spillover effect of regional health resource agglomeration capacity is no longer limited to geographically neighboring regions but manifests itself in cross-regional mobility as knowledge, talents, and technology flow across regions. Such cross-regional mobility makes the provincial (or municipal) health resource agglomeration capacity gradually shift from the technology-driven and demand-pulled linear pattern to the co-operative network pattern. In the co-operative network, the “visible hand” of the government and the “invisible hand” of the market jointly promote the intermingling of health resources between provinces (or municipalities) and influence the development of the co-operative network, enabling the health resource agglomeration capacities of different provinces (or municipalities) to show a dynamic, spatially correlated network structure. In this study, we performed social network analysis to analyze the spatial correlations between health resource agglomeration capacities of 31 Chinese provinces (or municipalities) from 2004 to 2018 and their influencing factors. This study helps deepen the understanding of the spatial correlations among health resource agglomeration capacities in China and provides reference for further implementation of the medical system reform and the rational allocation and utilization of health resources.

## 2. Methods and Data Sources

### 2.1. Methods

#### 2.1.1. Measurement of Health Resource Agglomeration Capacities

We used the entropy method and drew on the indicators determined by researchers [17] to measure health resource agglomeration capacities (Table 1). The entropy method mainly determines the objective weight according to the index variability. Generally speaking, the smaller the information entropy of an index is, the greater the degree of variation of the value of the index is, the more information is provided, and, the greater the role played in the comprehensive evaluation index system, the greater its weight is. On the contrary, the bigger the information entropy of an index is, the smaller the variation degree of the index value is, the less the information is provided, and, the smaller the role played in the evaluation system of the comprehensive index, the smaller its weight is. To avoid the impact of inconsistencies in the units of indicators on the calculation of the weight values, we employed the extreme difference method to standardize the data by transforming it within the range of 0 to 1.

The proportion (*y_ij_*) of sample *i* to total samples for the *j*th indicator can be calculated as follows (Equation (1)):

(1) $$y_{ij}=x_{ij}\overset{n}{\underset{i=1}{\sum }}x_{ij}\left(i\;=1,2,\ldots ,\;n;\;j\;=1,2,\ldots ,\;m\right),$$

where *n*, *m*, and *x_ij_* symbolize number of samples, number of indicators, and value of sample *i* for the *j*th indicator, respectively.

The information entropy (*e_j_*) of the *j*th indicator can be expressed as follows (Equation (2)):

(2) $$e_{j}=-k\overset{n}{\underset{i=1}{\sum }}y_{ij}\ln(y_{ij})$$

where *k* = 1/ln(*n*) (0 ≤ *e_j_* ≤ 1).

The difference coefficient (g_j_) of the *j*th indicator can be expressed as follows: *g*_j_ =1 − *e_j_*.

After normalizing the difference coefficients of indicators, the weight of the *j*th indicator can be obtained as follows (Equation (3)):

(3) ωaj=gj∑j=1mgj

Therefore, the Health Resource Agglomeration Capacities of a given area can be expressed as follows (Equation (4)):

(4) $${A=\sum_{j=1}^{m}\omega_{j}x_{ij}}_{\left(j\;=1,\;2,\;\ldots ,\;m\right)}$$

#### 2.1.2. Method for Constructing a Spatial Correlation Network of Health Resource Agglomeration Capacities

The spatial correlation network of inter-provincial (or inter-municipal) health resource agglomeration capacities is a collection of spatial correlations between provincial (or municipal) health resource agglomeration capacities. In the network diagram, each province (or municipality) is a “point,” and the spatial correlations between provinces (or municipalities) in health resource agglomeration capacity are “lines.” These points and lines form a spatial correlation network of the health resource agglomeration capacities at the provincial (or municipal) level. To construct such a spatial correlation network, we modified the traditional spatial gravity model by adding the geographical distance.

The traditional spatial gravity model is mainly used to measure the strength of the correlation between provinces (or municipalities). The commonly used equation for the measurement is as follows (Equation (5)):

(5) $$X_{e,f}=\lambda \frac{\sqrt{P_{e}G_{e}}\sqrt{P_{f}G_{f}}}{D_{ef}^{2}}$$

In Equation (5), *X_e,f_* denotes the strength of the correlation. *e and f* indicate two different provinces (or municipalities). *P_e_* and *P_f_* indicate the total populations of *e* and *f* at the end of the year, respectively. *G_e_* and *G_f_* represent the actual gross domestic product (GDP) of *e* and *f*, respectively. *D_e,f_* indicates the geographic distance between *e* and *f*. The square root of the product of the total population at the year end and the actual GDP of a province (or municipality) represents the actual economic condition of the province (or municipality). λ is a constant.

To reflect the spatial correlations between health resource agglomeration capacities more accurately, we drew on the modified gravity models applied by Nikzad [18], Teow et al. [19], Babri et al. [20], and Lundmark [21] to obtain our modified gravity model, as shown below (Equation (6)):

(6) $$X_{e,\;f}=\lambda_{e,f}\frac{\sqrt[{3}]{P_{e}N_{e}G_{e}}\sqrt[{3}]{P_{f}N_{f}G_{f}}}{\left[D_{f}/g_{e}-g_{f}\right]}$$

Equation (6) retains the total provincial (or municipal) populations at the end of the year, the actual provincial (or municipal) GDP, and the inter-provincial (or inter-municipal) geographic distance in the traditional gravity models. This equation also introduces the values of provincial (or municipal) health resource agglomeration capacity (i.e., *N_e_* and *N_f_*) and provincial (or municipal) GDP per capita (i.e., *g_e_* and *g_f_*) to measure the spatial correlation between health capital agglomeration capacities at the provincial (or municipal) level. The constant coefficient *λ_e,f_* denotes the proportion of the health resource agglomeration capacity of *e* in the total capacity of *e* and *f*.

Based on the modified gravity model, we can obtain the gravity matrix of the health resource agglomeration capacities of the 31 Chinese provinces (or municipalities). We calculated the average value of each row and column of the gravity matrix and recorded the average value of each row and column as a critical point. If the value of each cell of the gravity matrix is greater than or equal to the critical value, then a spatial correlation between the two provinces (or municipalities) exists, and the cell is recorded as 1. If the value of each cell of the gravity matrix is less than the critical value, then no spatial relationship between the two provinces (or municipalities) exists, and the cell is recorded as 0. Therefore, a spatial correlation network of health resource aggregation capacities can be constructed by transforming the gravity matrix into a spatial correlation matrix.

#### 2.1.3. Network Characterization of Spatial Correlations between Health Resource Agglomeration Capacities

The network density mainly measures the density of the correlation between provinces (or municipalities). The larger the network density, the closer the spatial correlation is between provinces (or municipalities). The value range of this measure is [0,1]. We let *NI* denote the network density, and *s* and *t* denote the actual number of correlations and the number of provinces (or municipalities) in the spatial correlation matrix, respectively. Then, we obtained the following equation (Equation (7)):

(7) NI=stt−12

The network level mainly describes to what extent provinces (or municipalities) are asymmetrically accessible to each other in the network, reflecting the dominant position of each province (or municipality) in the network. The larger the network level of a province (or municipality), the stronger its dominant role is in the network. The value range of this measure is [0,1]. We let *NR* denote the network level, and *α* and *β* denote the number of bidirectional and unidirectional correlations in the spatial correlation matrix, respectively. Then, we obtained the following equation (Equation (8)):

(8) $$NR=1-\frac{\alpha }{\alpha +\beta }$$

The network efficiency refers to the extent to which there are redundant lines in the network graph if the number of components contained in the known network graph is determined. Larger network efficiency indicates that the spatial connections between various provinces (or municipalities) are not close and that the spatial network structure is less stable and simpler. The value range of this measure is [0,1]. We let *NE* denote the network efficiency, *(t−1)* denote the minimum number of spatial correlations for all provinces (or municipalities), and *t (t−1)/2* denote the maximum number of spatial correlations for all provinces (or municipalities). Then, we obtained the following equation (Equation (9)):

(9) NE=1−s−t−1tt−12−t−1

The degree centrality mainly reflects the centrality of the health resource agglomeration capacity in the spatial network. A larger degree centrality value indicates that the province (or municipality) is more closely correlated with other provinces (or municipalities) and is more dominant in the spatial network. We used *PC* to represent the centrality of a point, and *l_1_*, *l_2_*, and *L* to represent the out-point degree (which refers to the number of correlations sent out by a province or municipality to other provinces or municipalities), in-point degree (which refers to the number of correlations received by a province or municipality from other provinces or municipalities), and the number of directed network nodes, respectively. Then, we obtained the following equation (Equation (10)):

(10) $$PC=(l_{1}+l_{2}/2L-2)$$

The betweenness centrality mainly reflects the degree of control that a province (or municipality) has over other provinces (or municipalities) in terms of the health resource agglomeration capacity. The more control it has over other provinces (or municipalities), the more important its mediator role becomes. We used *CC* to indicate the betweenness centrality, *L* to represent the number of directed network nodes, *S_jk_* to represent the number of shortcuts accessible between provinces (or municipalities) *j* and *k*, and *Z_jk_ (q)* to represent the probability of *q* being located on the accessible shortcuts between *j* and *k*. Then, we obtained the following equation (Equation (11)):

(11) $$CC=2\overset{L}{\underset{j}{\sum }}\overset{L}{\underset{k}{\sum }}z_{jk}\left(q\right)/\left(L^{2}-3L+2\right)\;z_{jk}\left(q\right)=s_{jk}\left(q\right)/s_{jk}\;j\neq k\neq q,j<k$$

The closeness centrality reflects the degree to which a province or municipality is not under the control of other provinces or municipalities in the spatial network of health resource agglomeration capacities. The larger the closeness centrality, the more independent the province or municipality is of other provinces or municipalities. We used *AC* to represent the closeness centrality, *L* to represent the number of directed network nodes, and *d_qj_* to represent the number of connecting lines between the shortcuts available between network nodes *q* and *j*. Then, we obtained the following equation (Equation (12)):

(12) $$AC=\frac{1}{\sum_{j}^{l}d_{aj}}$$

#### 2.1.4. Block Model Analysis for the Spatial Correlation Network of Health Resource Agglomeration Capacities

Block model analysis is a method for studying the network position model. Based on the block model theory, the role of each block in the spatial correlation network of health resource agglomeration capacities can be analyzed. There are usually four roles, namely the “net beneficial,” “net spillover,” “bidirectional spillover,” and “mediator” blocks. In the “net beneficial” block, the subject has more correlations within the block and receives more external correlations than it sends out. In the “net spillover” block, the subject has a relatively small number of internal correlations and sends out more external correlations than it receives. In the “bidirectional spillover” block, the subject sends out many correlations, both internally and externally. In the “mediator” block, there are a large number of correlations that the subject sends out and receives externally, while the number of internal correlations is lower.

### 2.2. Study Area and Data Sources

We selected 31 Chinese provinces (or municipalities) for the research, excluding Hong Kong, Macao, and Taiwan. Considering the continuity and stability of data, we merged the administrative regions according to the 2010 administrative division. As the statistics on medical and health care were incomplete before 2003, a large number of samples in the study area were missing, which was not conducive to analyzing the characteristics of the spatial correlation network. The 2003 SARS epidemic prompted China to systematically rethink the construction of public health resource systems. For these reasons, we selected 2004–2018 as the research period. The required data were mainly obtained from China Public Health Statistical Yearbooks, China Health and Family Planning Statistical Yearbooks, and China City Statistical Yearbooks from 2005 to 2019.

## 3. Spatial Correlation Analysis of China’s Health Resource Agglomeration Capacity

### 3.1. Spatial Distribution of the Health Resource Agglomeration Capacities in China

**Figure 1:**
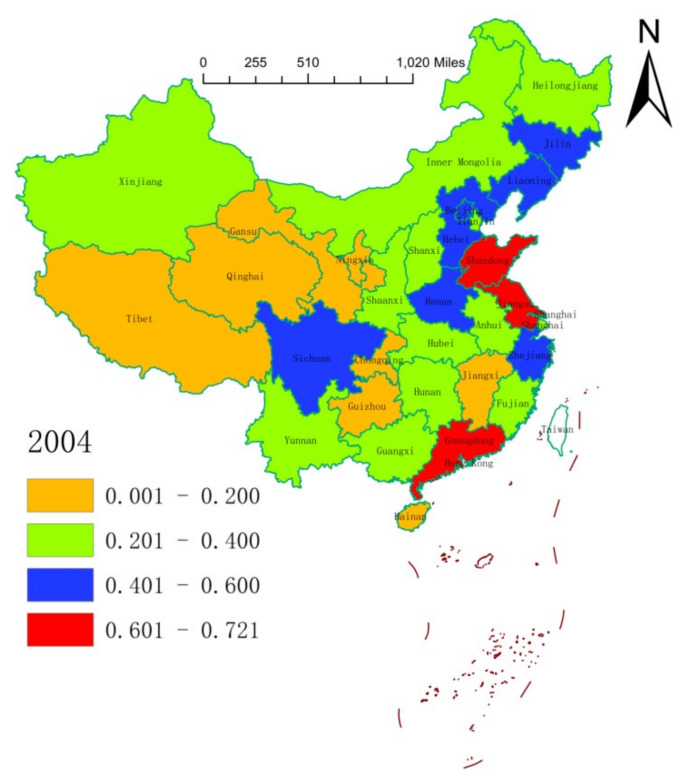
Spatial distribution of health resource agglomeration capacities in China in 2004.

**Figure 2:**
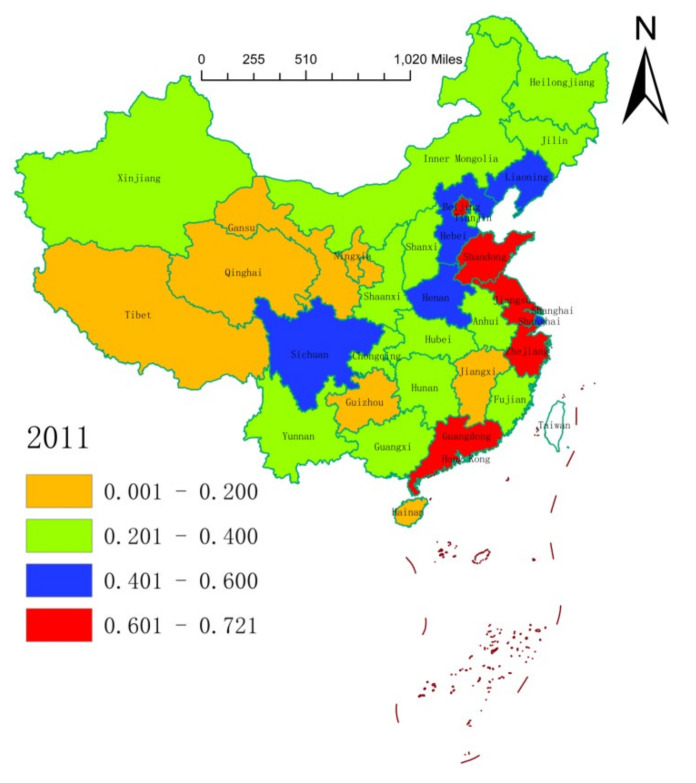
Spatial distribution of health resource agglomeration capacities in China in 2011.

**Figure 3:**
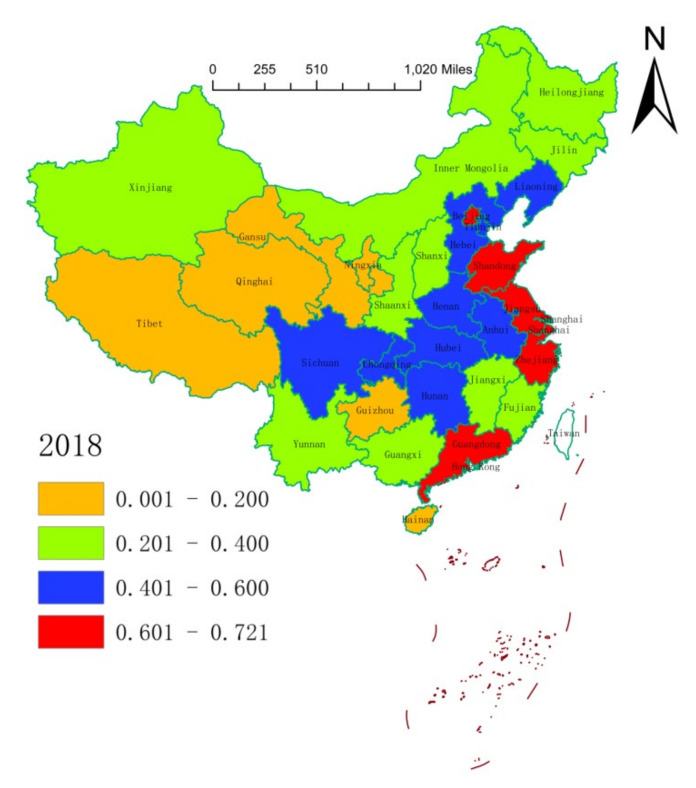
Spatial distribution of health resource agglomeration capacities in China in 2018.

We leveraged on the natural break classification method to plot the spatial distribution of the health resource agglomeration capacities of 31 Chinese provinces (or municipalities) in 2004, 2011, and 2018 (Figure 1, Figure 2 and Figure 3). On the whole, the health resource agglomeration capacities of 31 Chinese provinces (or municipalities) from 2004 to 2018 showed a gradual strengthening trend. At the local level, the health resource agglomeration capacities of Chinese provinces (or municipalities) showed a gradual weakening trend, with the strongest in the eastern region, the second strongest in the central region, and the weakest in the western region. One main reason for this situation is that deepening of the reform and opening-up of China, as well as continuous improvement of the market environment. Moreover, increasing government support and public demand for medical and health care in China have promoted local governments to rapidly improve their health resource agglomeration capacity. This situation can also be explained by the characteristics of the eastern, central, and western regions. That is, Zhejiang, Shanghai, Jiangsu, Shandong, Guangdong, and so on, which are located at the frontline of China’s reform and opening-up, have high levels of economic development and abundant government financial resources. This can attract abundant health resources to agglomerate. In addition, health resources, such as advanced technology, management experience, and cutting-edge equipment, are a priority with regard to agglomeration. Central provinces (or municipalities), such as Beijing, Hunan, Hubei, Anhui, and Jiangxi, have a large number of renowned and elite universities and possess quite a number of scientific research centers, engineering centers, and key laboratories, which provide a good scientific research platform for the agglomeration of health resources. Being economic centers and owing to their geographical locations and other advantages, central provinces (or municipalities) can also attract health resources to concentrate in the local area. Provinces (or municipalities) in the western region have a low level of economic development, a relatively scattered population distribution, generally high input costs for health resources, and low utilization efficiency of health resources, resulting in a lack of health resources and insufficient agglomeration capacity.

### 3.2. Structure of the Spatial Correlation Network of Health Resource Agglomeration Capacities in China

**Figure 4:**
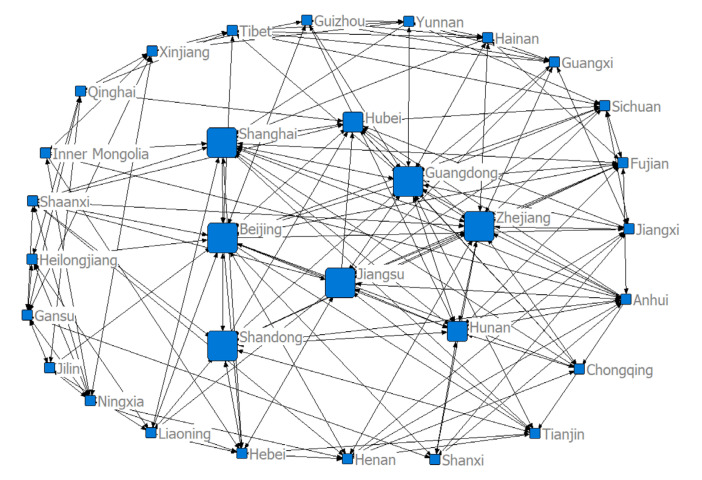
Structure of the spatial correlation network of health resource agglomeration capacities in China in 2004.

**Figure 5:**
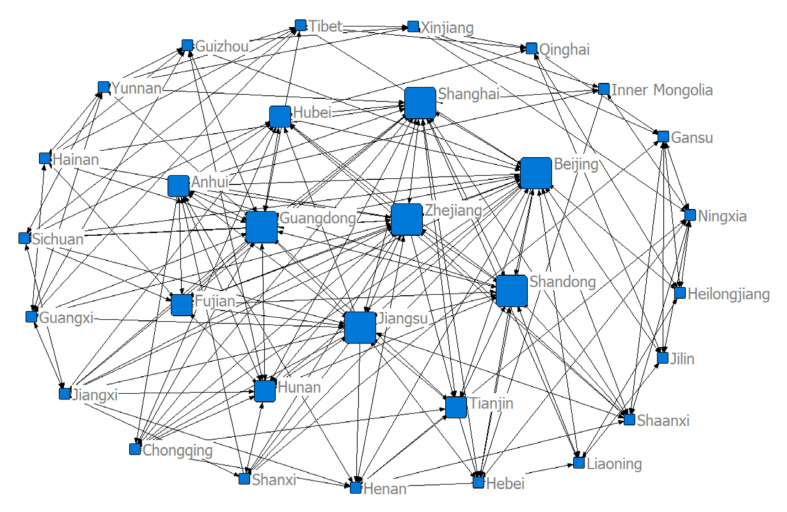
Structure of the spatial correlation network of health resource agglomeration capacities in China in 2011.

**Figure 6:**
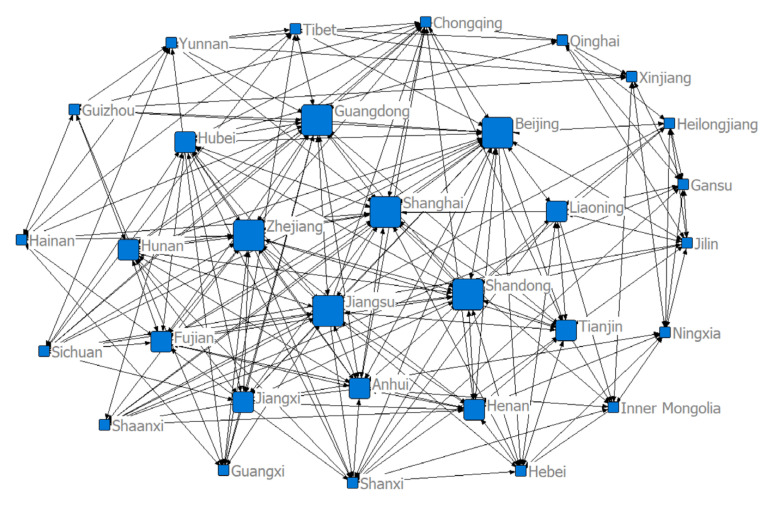
Structure of the spatial correlation network of health resource agglomeration capacities in China in 2018.

To investigate the spatial correlation network structure of China’s health resource agglomeration capacity, we constructed a matrix of spatial correlations based on a modified gravity model and used the visualization software NetDraw to draw a spatial network structure for the target sample in 2004, 2011, and 2018 (Figure 4, Figure 5 and Figure 6). We can see from the figures that there were eight larger directed nodes (provinces or municipalities) in the spatial network in 2004, 11 in 2011, and 14 in 2018. This indicates that these provinces (or municipalities) play greater roles, have more spatial correlations, and are at the center of the spatial network. This may be due to the fact that in the context of regional integration, health resources, such as practicing physicians, physician assistants, and professional nurses, are no longer limited to geographically neighboring regions, but rather manifest as trans-regional flows. The continuous development of marketization has further facilitated the free flow of health resources and accelerated the development of a spatial network of health resource agglomeration capacities. The economic development level, infrastructure construction level, and standard of living in provinces (or municipalities), such as the Beijing–Tianjin region, developed coastal areas, and the central region, are overall higher than those in other less-developed regions. The demand of residents for medical and health resources in these areas is even stronger. Therefore, local governments in developed regions need to invest a great deal of material, human, and financial resources in developing health resources to enhance these provinces’ (or municipalities’) ability to gather health resources and establish greater correlations with other provinces (or municipalities), so that they can play more prominent roles in the network.

### 3.3. Time Series Changes in the Structure of the Spatial Correlation Network of Health Resource Agglomeration Capacities in China

**Figure 7:**
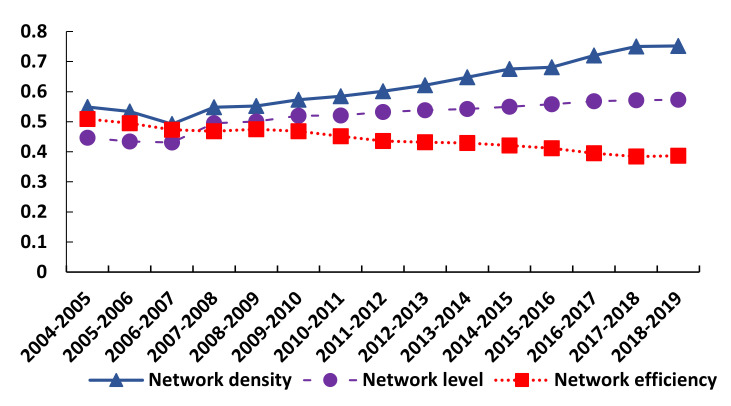
Time series changes in the structure of the spatial correlation network of health resource agglomeration capacities in China.

To explore the time series changes in the health resource aggregation capacities of 31 Chinese provinces (or municipalities), we calculated the network density, network level, and network efficiency from 2004 to 2018 based on Equations (7)–(9) (Figure 7). In this figure, the network density and level show an upward trend. Specifically, the network density increased from 0.549 in 2004 to 0.752 in 2018, and the network level rose from 0.447 in 2004 to 0.573 in 2018. This situation occurred mainly for two reasons. On the one hand, under the effect of marketization, all types of health resources are no longer limited to the local area but rather, break through geographic restrictions to realize cross-regional resource flows, thus strengthening spatial links. On the other hand, due to the general profit-seeking nature of resources, health resources, such as practicing physicians, physician assistants, and nurses, tend to gather preferentially in regions with higher levels of economic development or in developed coastal provinces (or municipalities), resulting in the dominant positions of these provinces (or municipalities) in the spatial network. The overall network efficiency showed a declining trend (from 0.509 in 2004 to 0.387 in 2018).The possible reason is that health resources are gradually agglomerating in economically developed provinces and cities, and the factor flow generated by this agglomeration is often only the flow of factor resources from underdeveloped provinces and cities to economically developed provinces and cities. This is a one-way connection. It is not a two-way connection, which weakens the connection passed from the economically developed provinces and cities to the economically underdeveloped provinces and cities, resulting in the weakening of the stability of the spatial network structure of China’s health resource aggregation capacities and further making the spatial network structure relatively rigid.

### 3.4. Centrality of the Spatial Correlation Network of Health Resource Agglomeration Capacities in China

Using Equations (10)–(12), we calculated the degree centrality, betweenness centrality, and closeness centrality of each province (or municipality) in the spatial correlation network of health resource aggregation capacities in 2004, 2011, and 2018 (Table 2). The table indicates that the high-ranking provinces (or municipalities) in terms of degree centrality, betweenness centrality, and closeness centrality are Beijing, Shanghai, Jiangsu, Zhejiang, Guangdong, Shandong, Hunan, Hubei, Fujian, Anhui, Jiangxi, and Tianjin; the low-ranking provinces (or municipalities) are Tibet, Qinghai, Gansu, Ningxia, Inner Mongolia, Heilongjiang, Yunnan, Guizhou, Xinjiang, Hainan, Shaanxi, and Shanxi. This means that the high-ranking provinces (or municipalities) have more spatial network connections with other provinces (or municipalities), and are closer to other provinces (or municipalities) in the network. Moreover, they are at the center of the network, and dominate the health resource agglomeration capacities of other provinces (or municipalities). Meanwhile, the provinces (or municipalities) at the bottom of the ranking have fewer spatial links with other provinces (or municipalities) and are farther away from other provinces (or municipalities), that is, marginal, in the network. Furthermore, their health resource agglomeration capacities are dominated in the network. Most of the high-ranking provinces (or municipalities) are in the central and eastern regions of China and have higher levels of economic development; thus, they are better than other provinces (or municipalities) in terms of human and material resources, financial resources, policy support, and so on, as well as in terms of the soft environment for health resource agglomeration. Consequently, they can attract health resources from neighboring provinces (or municipalities) to gather locally and establish spatial links with them more easily. The low-ranking provinces (or municipalities) are usually located on the periphery or in western China, where factors, such as inconvenient transportation, economic backwardness, and poor “soft” environmental conditions, constrain the improvement of their health resource agglomeration capacity. For this reason, they are dominated in the spatial network. In addition, the overall spatial network of health resource agglomeration capacities of 31 Chinese provinces (or municipalities) had an out-degree centrality of 61.23% and an in-degree centrality of 12.35%, indicating that there was a serious asymmetry in the spatial network of health resource agglomeration capacities of these provinces (or municipalities).

### 3.5. Block Model Analysis of the Spatial Correlation Network of Health Resource Agglomeration Capacities in China

Based on the networks shown in Figure 4, Figure 5 and Figure 6, we performed block model analysis for the spatial correlations between the health resource agglomeration capacities of 31 provinces (or municipalities) in China to reveal the relationship between spatial agglomeration and the blocks. We employed the CONCOR method (the CONCOR method is an iterative correlation convergence method, that is to say, by iterating and iterating the correlation between the rows and columns of the social network relation Matrix, the CONCOR method can converge and finally achieve the goal of grouping), with a maximum segmentation depth of 2 and a convergence criterion of 0.2. The health resource agglomeration capacities of 31 Chinese provinces (or municipalities) were divided into four blocks (Table 3). Block 1 consists of Beijing, Tianjin, Hebei, Shanxi, Inner Mongolia, Liaoning, Jilin, and Heilongjiang. Block 2 consists of Shanghai, Jiangsu, Zhejiang, Fujian, Shandong, Guangdong, and Guangxi. Block 3 consists of Anhui, Jiangxi, Hubei, and Hunan, Henan, Shaanxi, and Chongqing. Block 4 consists of Hainan, Sichuan, Guizhou, Yunnan, Tibet, Gansu, Qinghai, Ningxia, and Xinjiang.

**Figure 8:**
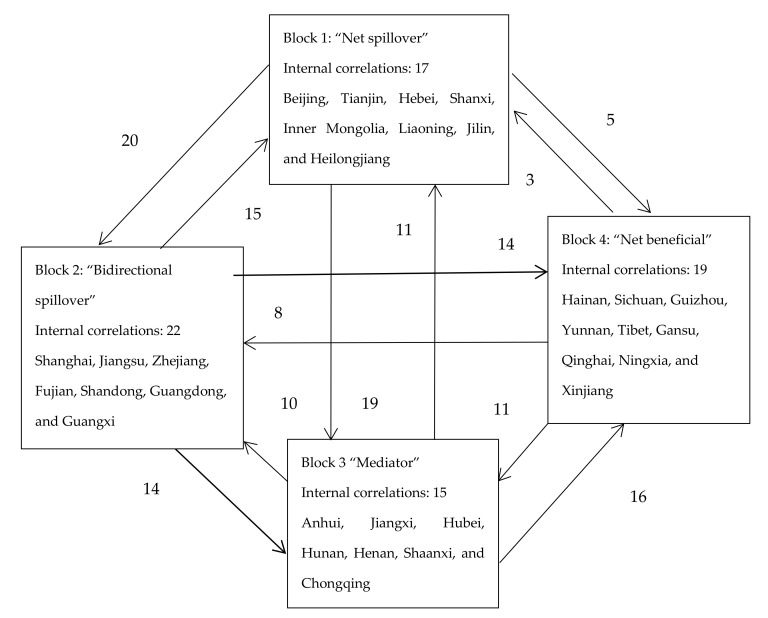
Correlations between the blocks of the spatial network of health resource aggregation capacities in China in 2018.

The characteristics of the four blocks are presented in Table 3. Block 1 sent out 44 correlations to and received 29 correlations from other blocks. It had 17 internal correlations, with its expected and actual proportions of internal correlations being 23.33% and 27.87%, respectively. Therefore, Block 1 was a “net spillover” block. This block sent out more correlations than it received and had fewer internal correlations, indicating that the spillover effects of block members were greater. Block 2 sent out 43 correlations to and received 38 correlations from other blocks. It had 22 internal correlations, with its expected and actual proportions of internal correlations being 20.00% and 33.85%, respectively. Therefore, Block 2 was a “bidirectional spillover” block. The correlations sent out and received by this block, as well as its internal correlations, were all greater in number, indicating that the members of this block exerted a bidirectional spillover effect on the health resource agglomeration capacities within and beyond this block. Block 3 sent out 37 correlations to and received 44 correlations from other blocks. It had 15 internal correlations, with an expected proportion of internal correlations being 20.00% and an actual proportion of internal correlations being 28.85%. Therefore, Block 3 was a “mediator” block. The numbers of correlations sent out and received by this block were relatively large. However, it had a few internal correlations. This characteristic shows that Block 3 played the role of “mediator” and “bridge” in the process of health resource agglomeration. Block 4 sent out 22 correlations to and received 35 correlations from other blocks. It had 19 internal correlations, with an expected proportion of internal correlations being 26.67% and an actual proportion of internal correlations being 46.34%. Therefore, Block 4 was a “net beneficial” block. It received more correlations from other blocks than it sent out and had fewer correlations, indicating a small spillover effect from the members of this block. As can be seen in Figure 8, each block had complex and close interactions with the other three blocks, in addition to their own internal correlations.

## 4. Analysis of Factors Influencing the Spatial Correlation Network of Health Resource Agglomeration Capacities in China

### 4.1. Selection of Influencing Factors

Learning from the research of scholars (Guo, et al., 2019) [17], we selected six factors influencing the spatial correlation network of health resource agglomeration capacities as follows. (1) Level of economic development: the higher the level of economic development, the stronger the residents’ demand for health resources. This factor is mainly measured by per capital GDP (PGDP). (2) Population size: the increase in population size can directly increase the pressure on the local government to improve its ability to concentrate health resources. This factor is mainly measured by the total population of the region at the end of the year (Pop). (3) Level of urbanization: an increase in the level of urbanization can lead to the scale development and intensification of health resources. This factor refers to the proportion of urbanization (Urb). (4) Level of education: students in institutions of higher education are the reserve army of health care personnel and the main support for the improvement of health resource agglomeration capacity. This factor is mainly measured by the number of students in regional institutions of higher education (Stu). (5) Wage level: the increase of wages in the health industry directly improves the work motivation of people engaged in the health industry and attracts health resources to gather. This factor mainly refers to the average wage level of employees in the health industry (Wag). (6) Level of financial health expenditure: the governments of economically developed regions are financially strong and invest more money in health resources. This factor mainly refers to the financial expenditure on the health industry (Exp).

Based on the analysis above, we constructed the following model:

R = f (PGDP, Pop, Urb, Stu, Wag, Exp).(13)

The equation above expresses the linkages between the data. We converted all data into the matrix form. We let R be the spatial correlation matrix of health resource agglomeration capacities of 31 Chinese provinces (or municipalities). R is the dependent variable, and the difference matrix of the six influencing factors (PGDP, Pop, Urb, Stu, Wag, and Exp) is the independent variable. Since the variables are a kind of relational data, and multicollinearity is prevalent in the data, the model cannot be tested for correlations using conventional methods. A quadratic assignment procedure (QAP), as a non-parametric, can analyze the relationship between matrices more robustly; thus, it is an important method in social network analysis.

### 4.2. Correlation Analysis of Factors Influencing the Spatial Network of Health Resource Agglomeration Capacities in China

In conducting QAP correlation analysis, we selected 8000 random substitutions to make the judgment. The results are listed in Table 4. The correlation coefficients of R with PGDP and Urb are 0.3012 and 0.2245, respectively. They are both significant at the 1% level, indicating that the level of economic development and urbanization of each province (or municipality) have a facilitating effect on the development of the spatial correlation network of health resource agglomeration capacities. Resources generally have a profit-seeking nature. Provinces (or municipalities) with higher levels of economic development are more likely to absorb the health resources of neighboring provinces (or municipalities), increasing the spatial correlations. The increase in the level of urbanization can connect previously scattered regions into one, breaking through the obstacles brought about by the administrative division of provinces (or municipalities). The correlation coefficients of R with Pop, Wag, and Exp are 0.2705, 0.1905, and 0.2265, respectively. They are all significant at the 5% level, indicating that the population size, wage level, and financial health expenditure level of each province (or municipality) are positively correlated with the increase in the spatial correlations of health resource agglomeration capacities. The increase in population size increases the pressure on local demand for health resources, prompting the government to increase health resources actively. A government’s strong finance and relatively greater investment in health resources also lead to higher wages in the health industry, thereby attracting health personnel to move across regions and increasing the spatial correlations. The correlation coefficient between R and Stu is 0.1204, but it is not significant.

## 5. Conclusions

The study built a spatial correlation network of health resource agglomeration capacities in China based on a modified spatial gravity model. The spatial correlations of health resource agglomeration capacities in China in 2004–2018 and their influencing factors were examined from the aspects of spatial distribution, network structure, time sequence changes, centrality, block model, and QAP correlation. The results showed that: (i) As a whole, 31 Chinese provinces (or municipalities) had a gradual increase in their health resource agglomeration capacity in 2004–2018; locally, a gradual decline was observed in the health resource agglomeration capacities of Chinese provinces (or municipalities), with the capacities being the strongest in the east, the second strongest in the central region, and the weakest in the west. (ii) The spatial network of health resource agglomeration capacities in China became increasingly dense; the network density and network level showed an upward trend, while the network efficiency showed an overall downward trend. (iii) The provinces (or municipalities) ranking high in-degree centrality, betweenness centrality, and closeness centrality mainly included Beijing, Shanghai, Jiangsu, Zhejiang, Guangdong, Shandong, Hunan, Hubei, Fujian, Anhui, Jiangxi, and Tianjin; the low-ranking provinces (or municipalities) mainly consisted of Tibet, Qinghai, Gansu, Ningxia, Inner Mongolia, Heilongjiang, Yunnan, Guizhou, Xinjiang, Hainan, Shaanxi, and Shanxi. (iv) Block 1, consisting of Beijing, Tianjin, Hebei, Shanxi, Inner Mongolia, Liaoning, Jilin, and Heilongjiang, had a net spillover effect in the spatial network of health resource agglomeration capacities; while Block 2, consisting of Shanghai, Jiangsu, Zhejiang, Fujian, Shandong, Guangdong, and Guangxi, had a bidirectional spillover effect in the network. Block 3, consisting of Anhui, Jiangxi, Hubei, Hunan, Henan, Shaanxi, and Chongqing, had a mediator effect in the network; and Block 4, consisting of Hainan, Sichuan, Guizhou, Yunnan, Tibet, Gansu, Qinghai, Ningxia, and Xinjiang, had a net beneficial effect in the network. (v) The level of economic development, level of urbanization, size of population, wage level, and level of financial health expenditure had a significant positive correlation with the spatial network of health resource agglomeration capacities.

This article uses the social network analysis method to analyze the spatial correlation characteristics of health resource agglomeration capacities in China. On this basis, we also use the QAP method to explore the influence factors of the spatial correlation network, but, due to the fact that the QAP method can only analyze the cross-section data, cannot analyze the panel data; thus, the next step is to improve or develop methods or software for analyzing the factors that influence spatial correlations.

### Policy Recommendations

To promote the healthy development of the spatial correlation network health resource agglomeration capacities of 31 provinces (or municipalities) more effectively and achieve proper allocation and utilization of health resources, we recommend the following countermeasures based on the abovementioned spatial correlation network and its influencing factors. (i) Enhance the radiation-driven role of core provinces (or municipalities) in health resource agglomeration. At present, Chinese provinces (or municipalities) with strong health resource agglomeration capacities are mainly concentrated in the eastern and central regions, the Beijing–Tianjin region, and the coastal areas, which are often at the center of the spatial network, and play a dominant role. Therefore, efforts should be made to promote actively the core provinces (or municipalities) to play a radiation-driven role in health resource accumulation capacity. On the one hand, after the core provinces (or municipalities) reach a certain scale of health resource agglomeration capacity, the waste of resources caused by the overcrowding of health resources should be prevented. To this end, part of their health resources can be transferred to neighboring provinces (or municipalities) and those with weaker health resource agglomeration capacities to drive neighboring provinces (or municipalities) to improve their health resource agglomeration capacities. On the other hand, the bidirectional exchange and position serving work should be properly carried out between core provinces (or municipalities) and other provinces (or municipalities), and active efforts should be made in convening some skills training courses and academic seminars. This can provide learning opportunities for personnel in provinces (or municipalities) with scarce health resources and improve their self-restoration skills in health resource agglomeration, ultimately forming a pattern of “agglomeration” nationwide. (ii) Rationally allocate health resources. With regard to the “club” effect in China’s ability to aggregate health resources and to narrow down the gap between regions in their ability to aggregate health resources, the Chinese government need to further decentralize its authority, establish a market-based, demand-oriented health resource transfer service platform, and create a favorable environment for the growth of health resources. The government also needs to remove institutional barriers actively to allow the flow of health resources, strengthen the construction of a health resource service system, and break the monopoly in the health resource agglomeration capacity. Specifically, the regional allocation and guidance policies for basic health resources should focus on the trends of population mobility and new types of urbanization to overcome gradually the urban–rural and regional divisions in public health resource services and establish a regular budget for the provision of basic health resources based on the resident population. In addition, it is necessary to give full play to the different roles and functions of the various blocks in the spatial network, and carry out bidirectional and precise regulation and control of health resources in the region. That is, while attention should be paid to the “bidirectional spillover” block to stimulate its “kinetic energy,” the “mediating” block should also be stimulated to strengthen further its transmission function. At the same time, barriers to the spatial interaction of health resources between the “net beneficial” and “net spillover” blocks should be eliminated to reduce the number of one-way links, while increasing the number of bidirectional links. (iii) Change the way of thinking in supporting public health resource services. The SARS outbreak in 2003 has led to a marked improvement in the ability of each Chinese region to aggregate medical and health resources. However, along with the gradual strengthening of the overall health resource agglomeration capacity, differences in the health resource agglomeration capacities of the eastern, central, and western regions have also been widening. In particular, since the outbreak of COVID-19 in Wuhan, China, issues, such as the non-equalization of health resource agglomeration capacities have once again caused the Chinese government to rethink its health resource agglomeration capacity. This requires the government to change its thinking on the support of medical and health resource services. That is, a shift should be made from full coverage support for basic medical and health resource services to focused support for regions lagging behind in medical and health resource services, as well as from a health-resource-allocation mechanism that relies on regional administrative levels to a health-resource-supply mechanism that relies on the social security system. On the one hand, it is necessary to improve the transfer payment system, increase the strength of financial support for health expenditures in backward regions, raise the level of urbanization and the wage level of medical and health personnel in backward regions, and expand the size of the provincial and municipal populations, so that health resources can be produced on a larger scale and intensity. On the other hand, a more open system for the management of medical and health resources should be established, for which social and foreign capitals can be fully utilized, and the potential of private hospitals and medical and health service providers can be released to break the rigidity and monopoly of the basic health resource supply system.

## Figures and Tables

**Table 1:** Indicator system and weights for evaluation of health resource agglomeration capacities.

	Indicator	Weight
Evaluation Indicators	Number of Hospitals	0.128
	Number of Community Health Service Centers/Stations	0.173
	Number of Certified Physician Assistants	0.080
	Number of Certified Physicians	0.084
	Number of Registered Nurses	0.075
	Number of Managers in Medical Institutions	0.080
	Number of Workers in Medical Institutions	0.082
	Number of Healthcare Practitioners / 1,000 People	0.088
	Total Assets of Health Institutions (RMB 1,000)	0.093
	Number of Hospital Beds / 1,000 People	0.117

**Table 2:** Centrality of the spatial correlation network of health resource agglomeration capacities in China.

Province or Municipality	2004			2011			2018		
	Degree Centrality	Betweenness Centrality	Closeness Centrality	Degree Centrality	Betweenness Centrality	Closeness Centrality	Degree Centrality	Betweenness Centrality	Closeness Centrality
Beijing	0.5	98.37	0.612	0.633	137.396	0.769	0.65	108.253	0.769
Tianjin	0.216	5.317	0.484	0.333	21.418	0.588	0.367	20.369	0.6
Hebei	0.233	16.459	0.484	0.267	15.605	0.508	0.283	13.529	0.526
Shanxi	0.150	13.326	0.484	0.183	19.621	0.536	0.283	17.175	0.536
Inner Mongolia	0.133	8.158	0.5	0.2	19.358	0.508	0.2	12.531	0.517
Liaoning	0.2	25.769	0.508	0.2	7.416	0.517	0.267	8.386	0.566
Jilin	0.15	7.405	0.484	0.183	11.353	0.508	0.25	15.426	0.556
Heilongjiang	0.15	7.405	0.484	0.167	9.046	0.508	0.217	8.59	0.536
Shanghai	0.4	50.962	0.6	0.458	47.906	0.638	0.517	43.681	0.667
Jiangsu	0.35	33.408	0.6	0.458	35.011	0.652	0.517	32.574	0.698
Zhejiang	0.433	41.043	0.577	0.533	45.835	0.682	0.55	38	0.682
Anhui	0.3	29.588	0.566	0.284	13.577	0.536	0.384	16.443	0.6
Fujian	0.316	12.606	0.536	0.333	9.997	0.536	0.383	10.603	0.556
Jiangxi	0.267	14.938	0.517	0.316	9.483	0.566	0.333	7.474	0.577
Shandong	0.317	23.705	0.6	0.417	24.51	0.6	0.467	31.974	0.625
Henan	0.317	30.353	0.545	0.3	21.461	0.556	0.333	19.728	0.577
Hubei	0.3	29.38	0.526	0.383	28.556	0.625	0.433	27.405	0.652
Hunan	0.4	33.194	0.577	0.367	16.258	0.577	0.384	11.851	0.577
Guangdong	0.517	69.654	0.612	0.533	62.078	0.652	0.567	54.294	0.682
Guangxi	0.25	9.257	0.526	0.233	6.974	0.508	0.25	6.321	0.508
Hainan	0.25	7.668	0.526	0.25	7.287	0.517	0.25	5.191	0.526
Chongqing	0.25	6.131	0.526	0.284	13.956	0.556	0.35	12.386	0.556
Sichuan	0.267	13.566	0.526	0.267	11.559	0.556	0.317	14.451	0.556
Guizhou	0.216	16.716	0.536	0.217	19.358	0.556	0.25	17.61	0.577
Yunnan	0.25	13.939	0.536	0.25	13.044	0.508	0.25	8.395	0.517
Tibet	0.267	33.671	0.556	0.217	21.096	0.492	0.267	25.743	0.577
Shaanxi	0.267	27.029	0.477	0.284	18.293	0.366	0.22	13.473	0.566
Gansu	0.233	24.873	0.5	0.217	17.25	0.5	0.284	13.843	0.517
Qinghai	0.2	26.005	0.462	0.217	23.835	0.545	0.217	15.67	0.556
Ningxia	0.267	40.32	0.517	0.217	14.998	0.476	0.267	21.142	0.536
Xinjiang	0.233	39.786	0.492	0.167	21.465	0.476	0.217	27.489	0.5

**Table 3:** Characteristics of each block in the spatial network of health resource aggregation capacities in China in 2018.

Block	Number of Correlations Received		Number of Correlations Sent		Expected Proportion of Internal Correlations (%)	Actual Proportion of Internal Correlations (%)
	Intra-block	Off-block	Intra-block	Off-block		
Block 1	17	29	17	44	23.33%	27.87%
Block 2	22	38	22	43	20.00%	33.85%
Block 3	15	44	15	37	20.00%	28.85%
Block 4	19	35	19	22	26.67%	46.34%

Note: The expected proportion of internal correlations = (Number of block members −1) / (Total number of cities −1). The actual proportion of internal correlations = Number of correlations sent between block members / Total number of correlations sent.

**Table 4:** Quadratic assignment procedure (QAP) correlation analysis of the influencing factors of spatial correlation network of health resource agglomeration capacities in China.

Influencing Factor	Correlation Coefficient	Significance Level	Standard Deviation	Minimum Value	Maximum Value
PGDP	0.3012	0.000	0.0012	−0.1803	0.3429
Pop	0.2705	0.011	0.0000	−0.1004	0.2906
Urb	0.2245	0.002	0.0026	−0.1107	0.3771
Stu	0.1204	0.110	0.0001	−0.2107	0.2005
Wag	0.1905	0.050	0.0006	−0.1503	0.2702
Exp	0.2265	0.013	0.0003	−0.1095	0.3045
